# Surgical and non-surgical management of spondylolisthesis: a comprehensive review

**DOI:** 10.25122/jml-2025-0039

**Published:** 2025-03

**Authors:** Dana-Georgiana Nedelea, Diana Elena Vulpe, Florentina Gherghiceanu, Bogdan Sorin Capitanu, Serban Dragosloveanu, Ioan Cristian Stoica

**Affiliations:** 1 Carol Davila University of Medicine and Pharmacy, Bucharest, Romania; 2 Department of Orthopedics, Foisor Clinical Hospital of Orthopedics, Traumatology and Osteoarticular Tuberculosis, Bucharest, Romania

**Keywords:** spondylolisthesis, conservative management, surgical treatment, spinal fusion, minimally invasive surgery, rehabilitation, spinal instability, ASD, Adjacent Segment Disease, COX, Cyclooxygenase, CT, Computed Tomography, MIS, Minimally Invasive Surgery, MRI, Magnetic Resonance Imaging, NSAIDs, Nonsteroidal Anti-inflammatory Drugs (typically used in the plural form), PLIF, Posterior Lumbar Interbody Fusion, ROM, Range of Motion, TLIF, Transforaminal Lumbar Interbody Fusion

## Abstract

Spondylolisthesis is a spinal condition characterized by the forward or backward displacement of a vertebral body, most commonly affecting the lower lumbar spine. It can be classified into different types, with isthmic and degenerative being the most prevalent. Early diagnosis is essential to initiate appropriate treatment based on symptom severity, degree of slippage, and neurological deficits. Non-surgical management is the first-line approach for low-grade spondylolisthesis (Grade I-II) and includes physical therapy, activity modification, pain management with nonsteroidal anti-inflammatory drugs or epidural steroid injections, and, in some cases, bracing. While most patients experience symptom relief with conservative treatment, those with progressive neurological deficits, severe pain, or significant instability may require surgery. Surgical options typically include decompression for nerve compression and fusion to stabilize the spine. The choice between decompression alone and decompression with fusion remains controversial, particularly in degenerative spondylolisthesis without initial instability. Posterior lumbar interbody fusion and transforaminal lumbar interbody fusion are the most performed techniques, with minimally invasive surgery gaining popularity due to its less aggressive impact on tissues and faster recovery. Long-term follow-up is necessary to monitor for complications such as adjacent segment disease, pseudarthrosis, or reoperation rate. Advances in imaging, surgical navigation, and regenerative medicine are important for the future of spondylolisthesis treatment, but current management remains centered on optimizing patient outcomes through individualized care and evidence-based treatment selection.

## INTRODUCTION

Spondylolisthesis is characterized by the displacement of a superior vertebral body relative to the inferior one in the sagittal plane. This displacement can occur in an anterior direction, known as anterolisthesis, in a posterior direction, referred to as retrolisthesis, or laterally [1,2]. Although spondylolisthesis can develop at any level of the vertebral column, it is most commonly observed in the lower lumbar spine. The condition may be acquired due to a combination of biomechanical stress and degenerative changes, or it can be congenital [3,4].

To better understand the mechanisms underlying spondylolisthesis, the Wiltse classification categorizes the condition into five distinct types: dysplastic (Type I), isthmic (Type II), degenerative (Type III), traumatic (Type IV), and pathologic (Type V). Isthmic and degenerative spondylolisthesis are the most frequent types and represent the primary causes of vertebral slippage [5].

Isthmic spondylolisthesis is primarily associated with a defect or fracture in the pars interarticularis, often caused by repetitive mechanical stress, and frequently progresses from a pre-existing spondylolysis [6]. This type is particularly common in young adults engaged in sports that involve repeated hyperextension of the lumbar spine, such as gymnastics or weightlifting. The incidence of isthmic spondylolisthesis in the general population is estimated to range between 4% and 8%, with a higher prevalence in males [7].

Degenerative spondylolisthesis, in contrast, is linked to the progressive deterioration of the facet joints and intervertebral discs, leading to vertebral instability over time. It is considered a condition associated with aging and is more frequently observed in adults, with a notable predominance in females, occurring up to six times more often than in males [4, 8].

Dysplastic spondylolisthesis results from congenital malformations affecting the neural arch, leading to vertebral instability [9,10]. Traumatic spondylolisthesis, another variant, results from fractures or dislocations of the posterior spinal elements, with the exception of the pars interarticularis [11]. Meanwhile, pathologic spondylolisthesis develops due to systemic or localized diseases that compromise vertebral integrity, such as neoplasms, infections, or metabolic bone disorders [12].

The severity of spondylolisthesis is commonly assessed using the Meyerding classification system, which grades the displacement of the superior vertebra in relation to the inferior vertebra. This system, determined through plain radiographs, was originally developed to evaluate anterolisthesis but can also be applied to cases of retrolisthesis. It categorizes the degree of slippage into five grades, ranging from grade I, representing less than 25% displacement, to grade V, or spondyloptosis, where vertebral slippage exceeds 100%. The severity of displacement is generally correlated with the intensity of symptoms, with higher-grade spondylolisthesis often associated with increased clinical manifestations [13].

The natural progression of spondylolisthesis varies depending on its etiology. Studies have reported an incidence rate of progression of 34% in degenerative spondylolisthesis, 32% in isthmic spondylolisthesis, and 4% in traumatic cases [14]. The condition may be asymptomatic or symptomatic, with clinical presentations ranging from localized back pain to more severe manifestations such as neurogenic claudication, radiculopathy, or, in extreme cases, cauda equina syndrome [3,15-17].

Management strategies for spondylolisthesis include both surgical and non-surgical approaches, though there remains ongoing debate regarding the most effective treatment modalities. The choice of intervention is largely dependent on symptom severity, the degree of vertebral slippage, and patient-specific factors. This study aims to review the existing literature and provide a comprehensive overview of current management approaches, focusing on patient treatment selection based on disease severity and clinical presentation.

## CLINICAL PRESENTATION AND DIAGNOSIS

### Symptoms and clinical evaluation

Not all patients with spondylolisthesis develop symptoms [18]. In many cases, low-grade spondylolisthesis (Grade I and II) does not cause significant clinical manifestations. However, higher-grade spondylolisthesis (Grade III and IV) is more frequently associated with symptoms, with reports indicating that 55% to 91% of these patients experience back pain, 44% to 55% develop radicular symptoms, and up to 50% report activity limitations [6,19,20]. Symptomatic patients often present with back pain, with specific characteristics depending on the affected region. The pain can develop gradually or appear acutely and is typically intermittent, manifesting mechanical characteristics [4,21]. It may worsen when transitioning from a supine to a standing position, with direct palpation over the affected vertebra, or during movements involving flexion or extension of the spine [22].

When spondylolisthesis leads to spinal nerve root compression, it can cause pain radiating along predictable dermatomal regions, depending on the level of slippage. Compression of the nerve roots in the lateral recess or neural foramen may result in symptoms such as paresthesia, sensory deficits, and weakness in the affected extremities [23-25]. In cases where spondylolisthesis is associated with lumbar spinal stenosis, symptoms develop gradually and may include myeloradiculopathy, muscle weakness, pathological reflexes, and neurogenic claudication [26]. Neurogenic claudication occurs due to central canal stenosis and is characterized by unilateral or bilateral discomfort, pain, weakness, and paresthesia in the thigh or calf, which worsens with walking or prolonged standing but improves with sitting and lumbar flexion. It is important to differentiate neurogenic claudication from vascular claudication, where symptoms are accentuated by walking and relieved by rest [27-29]. In progressive cases, spondylolisthesis can lead to bowel or bladder dysfunction, and in severe instances, it may result in cauda equina syndrome, requiring urgent medical intervention [30].

The physical evaluation of spondylolisthesis involves assessing posture, gait, spinal mobility, neurological function, and performing specific provocative tests to determine vertebral instability and nerve involvement. At examination, patients may present with an anteriorly shifted posture, increased lumbar lordosis, or a visible step-off deformity in cases of significant vertebral slippage [31,32].

Gait analysis can reveal an unsteady pattern or a shortened pace, particularly in high-grade spondylolisthesis. Range of motion (ROM) is typically restricted in lumbar extension, while flexion may provide symptom relief, especially in degenerative spondylolisthesis [16, 33].

Neurological examination assesses motor strength, sensory deficits, and reflex changes, with weakness or diminished reflexes suggesting radiculopathy. Special tests such as the Stork test, where the patient stands on one leg and extends the lumbar spine, may accentuate pain in cases of pars defects [34]. The straight leg raise test can help identify nerve root irritation when pain radiates down the leg. In high-grade cases, the Phalen-Dickson sign, where patients adopt a posture with hip and knee flexion while standing, may be observed as a compensatory mechanism [35-37].

Differentiating neurogenic and vascular claudication can be guided by the bicycle test, where patients with neurogenic claudication secondary to spondylolisthesis may tolerate prolonged cycling in a flexed position, whereas vascular claudication remains activity-dependent [38].

### Imaging modalities

Plain radiographs in anteroposterior and lateral views serve as the first-line imaging modality for evaluating spondylolisthesis due to their rapid availability, accessibility, and cost-effectiveness [25,39,40]. These images allow for the assessment of vertebral alignment, spinal instability, and classification based on the Meyerding grading system. This system quantifies the degree of anterior translation of the slipped vertebra relative to the adjacent one, categorizing it into five grades: grade I (0–25%), grade II (26–50%), grade III (51–75%), grade IV (76–100%), and grade V (>100%) [13,41].

Standing (erect) and supine X-rays provide different perspectives on spondylolisthesis grade due to spinal loading and positioning variations. In the standing position, gravity and patient weight-bearing can accentuate vertebral displacement, making it a more reliable assessment for detecting instability or progression of the slip. In contrast, supine X-rays may underestimate the degree of spondylolisthesis as gravitational forces are reduced, leading to potential underdiagnosis of dynamic instability. Therefore, standing radiographs are preferred for evaluating the true extent of spondylolisthesis and assessing its functional impact [42,43].

Standing anteroposterior and lateral radiographs of the entire spine are recommended in patients presenting with localized pain, clinical deformity, or red flag symptoms such as cauda equina syndrome, suspected malignancy, infection, or fracture [44,45]. Additionally, dynamic flexion-extension radiographs are valuable in assessing segmental stability, with instability defined by a change in disc angle greater than 10 degrees or a translation shift exceeding 3 mm when comparing static and dynamic views [46]. While oblique radiographs have historically been considered more sensitive than standard anteroposterior and lateral views in detecting pars interarticularis defects, their routine use is not justified due to the increased cost and radiation exposure, which do not significantly improve diagnostic accuracy [47-49].

If plain radiographs show normal results but clinical suspicion remains, computed tomography (CT) represents an alternative for detecting defects and bony abnormalities, particularly at the level of the pars interarticularis [50]. CT is also an essential tool for preoperative planning, helping in the assessment of pedicle screw size and allowing for three-dimensional reconstruction of the spine. Additionally, single-photon emission computed tomography can be utilized to identify stress reactions at the pars level by detecting areas of increased radiotracer uptake [51].

Magnetic resonance imaging (MRI) is primarily indicated for patients with neurological deficits, as it provides detailed visualization of neural structures, disc degeneration, and other soft tissue pathologies. However, due to its low predictive value in diagnosing spondylolisthesis, MRI is not typically used as a first-line imaging modality [52-54]. Unlike X-rays, which provide dynamic or weight-bearing views, MRI is performed in a supine, unloaded position, which may not accurately reflect the degree of vertebral slippage under normal physiological conditions. As a result, standing or flexion-extension radiographs are preferred for initial assessment, as they better illustrate spinal instability and mechanical load effects [55].

## NON-SURGICAL MANAGEMENT OF SPONDYLOLISTHESIS

### Indications for conservative treatment

When selecting a management strategy for spondylolisthesis, several factors must be considered, including the degree of slippage, underlying etiology, symptom severity, and patient-specific characteristics such as age, sex, and activity level [56,57]. Early detection and prompt intervention can improve clinical outcomes. Conservative treatment is typically recommended for patients with low-grade spondylolisthesis (grades I and II), especially those who are asymptomatic or have mild to moderate symptoms without significant neurological deficits [39]. This approach is also suitable for young athletes with spondylolysis or early-stage spondylolisthesis, as well as adults with degenerative spondylolisthesis who do not present progressive neurological decline [25, 58-60]. For individuals with mild neurogenic claudication due to spinal stenosis associated with spondylolisthesis, conservative management is considered if imaging does not indicate rapid disease progression and symptoms remain stable [61]. Although there is no clear consensus regarding the management of high-grade spondylolisthesis (grade III or IV), some evidence suggests that asymptomatic or mildly symptomatic patients may also benefit from conservative treatment [62, 63]. A study by Lundine *et al*. on high-grade spondylolisthesis patients found that observation alone did not accelerate disease progression in asymptomatic individuals, and delayed surgical intervention did not result in worse clinical outcomes [64].

Regular follow-up is essential for patients undergoing conservative treatment to monitor both clinical and imaging progression. The most generally accepted protocol begins with one to two days of rest and activity modification, followed by administering analgesics or anti-inflammatory medication. If symptoms persist beyond 1 to 2 weeks, physical therapy is typically introduced as the next step [65,66].

### Pain management

Pain management requires careful attention when selecting the appropriate medication for each patient. The first-line treatment for lower back pain associated with spondylolisthesis includes nonsteroidal anti-inflammatory drugs (NSAIDs) and/or analgesics [3,67]. NSAIDs provide both analgesic and anti-inflammatory effects depending on the dosage. Lower doses primarily help with mild pain relief, whereas higher doses are necessary to control inflammation contributing to nerve irritation and mechanical discomfort [68,69]. Due to the high prevalence of degenerative spondylolisthesis in the elderly population, where multiple comorbidities may be present, NSAIDs should be prescribed with caution. Traditional NSAIDs such as ibuprofen and naproxen, which inhibit both cyclooxygenase (COX)-1 and COX-2, are associated with an increased risk of gastrointestinal and renal adverse effects when used long-term [70]. On the other hand, selective COX-2 inhibitors like celecoxib are related to a higher risk of cardiovascular complications [71,72].

A review by Pepijn *et al*. concluded that NSAIDs are not significantly more effective than other analgesics, such as acetaminophen, opioids, or muscle relaxants, in treating lower back pain [73]. Given this finding, along with the potential adverse effects of NSAIDs, acetaminophen may be considered as an initial treatment option [74]. If symptoms persist, NSAIDs may be introduced cautiously. In cases of more severe pain, muscle relaxants may be used to reduce muscle spasms, while opioids are reserved for short-term use in patients whose pain is unresponsive to other treatments. Although these medication classes do not necessarily provide superior efficacy compared to acetaminophen or NSAIDs, no strong evidence suggests they should be avoided [75].

Epidural steroid injections are indicated in patients with symptomatic spondylolisthesis experiencing nerve root irritation, neurogenic claudication due to spinal stenosis, persistent pain despite NSAID use, physical therapy, and activity modification, or those who require temporary pain relief until surgery [76,77]. These injections deliver corticosteroids such as methylprednisolone into the epidural space to reduce inflammation and relieve pain. Studies have demonstrated short-term benefits, including pain reduction and functional improvement, but long-term efficacy remains variable [78-80].

### Physical therapy and rehabilitation

Physical therapy and rehabilitation play an important role in the conservative management of spondylolisthesis. According to the World Federation of Neurosurgical Societies Spine Committee recommendations, a structured physical therapy program for at least 3 weeks is beneficial for patients meeting the criteria for nonoperative treatment [81]. It is typically initiated when rest and medication fail to relieve symptoms. The therapy plan should be individualized based on the etiology of spondylolisthesis and the patient's activity level [82,83].

For patients with isthmic spondylolisthesis, who are often young and physically active, including athletes, the primary goal of physical therapy is to restore their previous physical condition and enable a safe return to activity [58,84]. Rehabilitation programs may focus on core stability, strength recovery, resistance training, postural correction, and flexibility exercises. The primary objectives of physical therapy are to reduce pain, improve ROM, and enhance strength and spinal stability [82,85].

If there are no signs of symptom improvement after completing at least four to six weeks of physical therapy course, it is advisable to consider alternative treatment options. Further assessment may be required to determine whether additional conservative measures, interventional pain management, or surgical intervention should be considered [86].

#### Bracing

Bracing is sometimes utilized as part of the conservative treatment of spondylolisthesis, particularly in cases of low-grade slips (grade I and II) and in pediatric or adolescent patients experiencing symptomatic instability. The primary objectives of bracing are to restrict excessive spinal motion, reduce mechanical stress on the affected vertebrae, and reduce pain [82,87].

Bracing is usually recommended for acute spondylolysis or early-stage spondylolisthesis in young athletes, especially in sports that involve repeated lumbar hyperextension, such as gymnastics or football [82]. A study conducted by Bell *et al*. examined adolescents with grade I or II isthmic spondylolisthesis who underwent bracing treatment for 25 months. The findings demonstrated reduced pain and no evidence of further vertebral slippage [88].

In adults with degenerative spondylolisthesis, bracing is less frequently used due to the chronic nature of the condition and the lack of strong evidence supporting its long-term benefits. Additionally, long-term bracing in adults may lead to muscle deconditioning and does not address the underlying degenerative pathology, which may explain why studies have shown minimal benefit in this population [89]. A systematic review by Garet *et al*. found that only one out of ten included studies examining degenerative spondylolisthesis reported bracing as an effective intervention [90].

#### Flexion/extension strengthening exercises

Flexion and extension strengthening exercises enhance core stability and improve the function of muscles that support the spine while minimizing movements that may worsen the condition [91]. Flexion exercises, such as pelvic tilts and seated abdominal contractions, primarily target the anterior core muscles, helping reduce lumbar lordosis and stress on the affected vertebrae [92]. Carefully controlled extension exercises, such as modified back extensions and bird-dog variations, strengthen the lumbar extensor muscles, which contribute to maintaining proper spinal alignment. These exercises should be performed in a controlled manner, avoiding excessive hyperextension and ensuring close monitoring by a physical therapist [93].

A study conducted by Sinaki *et al*. divided patients with back pain due to spondylolisthesis into two groups, one performing flexion exercises and the other performing extension exercises. After 3 months, the recovery rate was 58% in the flexion group compared to only 6% in the extension group [94]. This difference may be explained by the posture-dependent compression of the spinal canal, where extension results in canal narrowing, while flexion promotes canal widening and reduces nerve root compression [95].

In addition to flexion and extension exercises, endurance training is also beneficial for patients, as many report that their walking and standing capacity is affected. Therefore, a walking-based training program should be initiated, starting with slow walking and gradually increasing speed. In younger patients, this may also include running as part of their rehabilitation [28,96].

#### Stabilization exercises

Stabilization exercises focus on improving core strength, spinal alignment, and neuromuscular control while reducing pain and preventing further slippage. These exercises target deep stabilizing muscles such as the transverse abdominis, multifidus, pelvic floor, and gluteal muscles, which provide support to the lumbar spine [97,98]. O'Sullivan proposed a training approach that engages these stabilizing muscles both at rest and during movement while minimizing strain on superficial muscles [99]. While strengthening deep abdominal muscles is essential, addressing the lumbar multifidus is also important, as studies have demonstrated its role in core stabilization. Initially, exercises are performed in lying, seated, and standing positions, gradually progressing to unstable conditions to enhance neuromuscular control [100,101].

O'Sullivan *et al*. demonstrated that patients with chronic lower back pain who followed a stabilization program incorporating deep abdominal muscle and multifidus training experienced reduced pain levels and improved physical function [102]. Another essential aspect of stabilization exercises is addressing muscle imbalances, as poor balance can contribute to postural deficits and disability. If left uncorrected, these imbalances can lead to further functional impairments and suboptimal rehabilitation outcomes [28,103].

#### Spinal manipulation

Spinal manipulation is a manual therapy technique used for various spinal disorders, though its role in the management of spondylolisthesis remains controversial. Spinal manipulation involves the application of controlled force to specific joints of the spine to restore movement, reduce pain, and improve function. This technique may include high-velocity, low-amplitude thrusts or more gentle mobilization methods to enhance spinal alignment and mobility [104]. In low-grade spondylolisthesis (grades I and II) without neurological deficits, gentle spinal mobilization techniques may relieve pain and improve spinal flexibility [82,105]. However, high-velocity, low-amplitude (HVLA) manipulations involving rapid thrusting movements are generally discouraged due to the potential risk of worsening vertebral instability and symptoms [106]. A study by Mierau *et al*. found that patients with spondylolisthesis who received spinal manipulation techniques did not show significant long-term benefits, additionally supporting the cautious application of this approach in the management of spondylolisthesis [107].

### Lifestyle modifications

Lifestyle modifications are complementary to other treatment approaches. As previously mentioned, the initial step in conservative management typically involves 1 to 2 days of rest with activity restriction. These modifications focus on activity adjustments, weight management, ergonomic improvements, and overall spinal health to help alleviate symptoms and prevent further progression of the condition [108,109].

Activity modification, including avoiding activities that place excessive strain on the lower back, such as repetitive lumbar hyperextension, heavy lifting, or high-impact sports, can help minimize pain and reduce the risk of worsening vertebral slippage. Instead, patients are encouraged to participate in low-impact exercises like swimming, cycling, or walking, which provide cardiovascular benefits while placing minimal stress on the spine [110].

Weight management is another important consideration, as excess body weight increases mechanical stress on the lumbar spine, contributing to pain and disease progression [111]. A meta-analysis by Rahman *et al*., which included 33 studies, found a strong correlation between overweight, obesity, and an increased risk of low back pain [112]. Ergonomic modifications in daily activities and posture can also be beneficial. Patients are advised to maintain proper spinal alignment when sitting, standing, or lifting objects to reduce lower back strain [113]. Prolonged sitting has been associated with worsening symptoms, and according to a study by Wong *et al*., regular movement is recommended every 20 minutes to prevent discomfort and maintain spinal health [114]. By integrating these lifestyle modifications, patients with spondylolisthesis can better manage their symptoms, improve their overall functional capacity, and reduce the risk of disease progression.

### Evidence and outcomes

Patients eligible for non-surgical treatment are advised to follow a conservative management program for at least 6 months to evaluate its effectiveness. Studies suggest that only 10-15% of these patients will eventually require surgical intervention [1,115]. The natural progression of the disease was assessed in a study by Matsunaga *et al*., which followed 145 patients with degenerative spondylolisthesis who underwent non-surgical treatment for a minimum of 10 years. Progressive slippage was observed in 49 patients (34%), while from 110 patients who initially had no neurological deficits, 84 (76%) remained neurologically intact after a decade [14].

For patients with isthmic spondylolisthesis, conservative treatment for three to six months has demonstrated good outcomes, managing most unilateral pars lesions and approximately 50% of bilateral lesions [59]. Once pain declines, athletes may resume sports activities, but modifications should be made to prevent further disease progression [116]. A review by Bouras *et al*. concluded that 70-90% of athletes with spondylolisthesis can return to athletic participation within three to 6 months following conservative treatment [39].

The success of conservative management is influenced by the severity of initial slippage. In patients with grade I or II spondylolisthesis, pain relief was achieved in 69% of cases, whereas only one out of 12 patients with grade III or IV experienced adequate symptom relief [110,117].

A thorough evaluation is essential to determine whether a patient is a suitable candidate for conservative treatment. Additionally, for those with high-grade spondylolisthesis but mild symptoms, delaying surgery may be beneficial; however, careful monitoring is required to assess disease progression and symptom severity over time [6].

## SURGICAL MANAGEMENT FOR SPONDYLOLISTHESIS

### Indications for surgery

As previously mentioned, 10-15% of patients initially managed non-surgically fail to respond to conservative treatment and continue to experience persistent and debilitating symptoms, ultimately requiring surgical intervention. The decision to proceed with surgery is based on symptom severity, functional limitations, and radiographic findings rather than the degree of vertebral slippage alone [25].

A review by Herkowitz *et al*. concluded that candidates for surgery include patients with persistent or recurrent back pain that significantly affects their quality of life even though they followed at least three to six months of non-operative treatment [65]. Additional indications for surgical intervention include worsening radiculopathy, progressive muscle weakness, sensory loss, or severe neurogenic claudication that does not improve with conservative measures [118].

In more urgent cases, spondylolisthesis leading to cauda equina syndrome necessitates immediate surgical decompression to prevent permanent neurological damage. Furthermore, patients with significant spinal instability, as demonstrated by dynamic imaging (defined as >3 mm difference and >10 degrees change in disc angle when comparing static with dynamic imaging), are more likely to require surgical intervention to restore spinal alignment and prevent further deterioration [119,120].

### Surgical techniques

#### Decompression alone vs. decompression with fusion

Surgical management of spondylolisthesis typically involves either decompression alone in cases where nerve root or spinal cord compression is the primary concern or decompression combined with spinal fusion to restore stability and prevent further slippage [121]. The primary decompression techniques used are laminectomy, which relieves spinal canal stenosis, and foraminotomy, which reduces nerve root compression [122]. Fusion is generally recommended alongside decompression for patients with significant spinal instability, particularly in high-grade spondylolisthesis (Grade III or IV), which is more commonly associated with isthmic rather than degenerative spondylolisthesis [123-125].

While surgery is effective in reducing symptoms and clear indications exist for fusion in unstable cases, controversy persists regarding the optimal approach for managing low-grade (Grade I and II) degenerative spondylolisthesis without radiographic instability [126]. The debate focuses on whether decompression alone is sufficient or if fusion should be routinely added. Some studies suggest that decompression alone provides comparable long-term outcomes while reducing surgical costs, hospital stays, and the risk of postoperative complications associated with fusion [127-133]. However, other research indicates that decompression alone can contribute to postoperative spinal instability, particularly in cases where extensive bone removal is necessary, thus increasing the risk of further slippage over time [130,134-138].

In past decades, decompression with fusion was widely considered the standard approach, accounting for over 90% of spondylolisthesis surgeries in some countries. However, recent studies have questioned this practice, with multiple trials and reviews generating conflicting results [137,139]. A systematic review by Gadjradj *et al*., which analyzed seven high-quality studies comparing decompression alone to decompression with fusion in patients with low-grade spondylolisthesis, found no significant advantages of fusion in terms of pain relief, functional outcomes, or reoperation rates. Additionally, decompression alone was associated with lower intraoperative blood loss, reduced complication rates, and shorter hospital stays [132].

Similarly, a randomized controlled trial by Forsth *et al*., involving 247 patients with lumbar spinal stenosis and spondylolisthesis, reported no long-term benefits of fusion over decompression alone at 2 years post-surgery [140]. Another randomized trial by Austevoll *et al*., which included 267 patients, confirmed that decompression alone was non-inferior to decompression with fusion, with no significant differences in pain, function, or quality of life between the two groups [133]. However, a smaller randomized trial by Ghogawala *et al*., involving 66 patients, reported a slight improvement in health-related quality of life at 2 years for patients who underwent fusion alongside decompression, although these patients had a higher reoperation rate (34% vs. 12% in the decompression-only group) [138].

In contrast to degenerative spondylolisthesis, managing isthmic spondylolisthesis is generally more straightforward, with fusion being necessary due to the structural instability resulting from the pars defect [141-144]. Decompression alone is rarely sufficient, as it may exacerbate vertebral slippage and lead to worse outcomes. However, one area of debate concerns whether fusion is required in low-grade, stable cases. While evidence suggests that decompression alone may be appropriate for carefully selected patients, most studies favor the combined approach of decompression with fusion [141,145,146].

Another debate area in the surgical treatment of isthmic spondylolisthesis is whether reduction of the slipped vertebra before fusion provides better outcomes than in situ fusion without reduction [147,148]. Some concerns exist that reducing the slippage may stretch the lumbosacral nerve roots, increasing the risk of postoperative neurological complications [149, 150]. A meta-analysis comparing these approaches found that both in situ fusion and reduction followed by fusion resulted in favorable clinical outcomes. However, reduction was associated with a higher fusion rate, improved radiographic alignment, and shorter hospital stays without significantly increasing neurological risks [151].

Ultimately, whether treating degenerative or isthmic spondylolisthesis, the decision to perform fusion must be individualized based on patient-specific factors, including the degree of slippage, symptoms, and the potential risks of each surgical approach. Further large-scale, well-controlled studies are needed to establish clearer guidelines and reach a consensus on the optimal treatment strategy, as current opinions remain divided.

#### Posterior lumbar interbody fusion (PLIF) vs. transforaminal lumbar interbody fusion (TLIF)

Posterior lumbar interbody fusion (PLIF) and transforaminal lumbar interbody fusion (TLIF) are two widely utilized surgical techniques for stabilizing the spine in patients with spondylolisthesis. Both procedures involve removing the intervertebral disc and inserting an interbody graft or cage to facilitate fusion between the affected vertebrae. Their primary objectives are to restore spinal alignment, prevent further slippage, and reduce nerve compression associated with spondylolisthesis [152].

PLIF is performed through a bilateral posterior approach. This method allows for the placement of interbody grafts on both sides of the disc space and is often suitable for degenerative cases requiring spinal fusion [153]. However, PLIF involves significant nerve root retraction, which may raise the risk of neurological complications. Furthermore, the extensive exposure required for this technique leads to greater blood loss and soft tissue disruption, potentially delaying recovery [154,155]. PLIF is generally indicated for cases where bilateral nerve compression is present or when a larger fusion surface is necessary for long-term spinal stability [156].

TLIF, in contrast, is performed through a unilateral approach, accessing the disc space via the foraminal window. By preserving one side of the posterior spinal structures, TLIF reduces the degree of nerve retraction and reduces the risk of postoperative neurological deficits compared to PLIF [157,158]. The interbody graft in TLIF is placed at a slightly more oblique angle than in PLIF, which may bring challenges in correcting coronal imbalance and restoring lordosis [159,160]. Despite these limitations, TLIF remains an effective option for spinal stabilization and is frequently recommended for patients with unilateral radiculopathy caused by foraminal stenosis or degenerative changes associated with spondylolisthesis [152].

Several studies have compared these techniques to assess their efficacy and associated complications. Humphreys *et al*. examined patients treated with PLIF and TLIF, reporting a higher incidence of complications in the PLIF group, including graft malpositioning in four cases, whereas no such complications were observed in the TLIF group [159]. Additionally, multiple studies have indicated that PLIF is associated with significantly greater operative time and blood loss than TLIF. However, despite these differences, both techniques have demonstrated comparable effectiveness in achieving solid spinal fusion and slip reduction [161-164]. In addition to these findings, a systematic review and meta-analysis by Zhang *et al*. analyzed seven studies and concluded that while PLIF was linked to a higher complication rate and longer operative time, there was no significant difference between PLIF and TLIF in terms of clinical outcomes, patient satisfaction, or radiographic fusion rates [165].

#### Minimally invasive surgery (MIS) techniques

Minimally invasive surgery (MIS) techniques have gained popularity in the treatment of spondylolisthesis due to their ability to produce similar outcomes to traditional approaches while reducing soft tissue damage, blood loss, and postoperative complications. Traditional open procedures, such as PLIF and TLIF, often require extensive muscle dissection, leading to delayed recovery and an increased risk of complications. In contrast, MIS approaches focus on preserving surrounding musculature, reducing operative time, and improving overall patient outcomes [166,167].

One of the most commonly used MIS techniques for spondylolisthesis is MIS-TLIF, which employs a unilateral paramedian approach through a small incision [168]. A prospective study analyzing 345 patients across multiple institutions demonstrated that both MIS and open surgery are effective in reducing pain and restoring function, with comparable surgical and functional outcomes [169]. A systematic review and meta-analysis of 10 studies further confirmed that while MIS and open techniques provided similar pain relief and functional improvement, MIS was associated with reduced blood loss but slightly longer operative times due to its technical complexity. Additionally, although hospital stays were similar between the two groups, a greater proportion of MIS patients returned to work earlier [170].

Endoscopic decompression techniques, such as endoscopic laminectomy or foraminotomy, involve small working channels that allow targeted removal of compressive structures with reduced surgical trauma and faster postoperative recovery [171]. Research by Sharma *et al*. supports the advantages of this technique, with improvements in patient-reported outcomes such as Oswestry Disability Index, Visual Analogue Scale, and Patient-Reported Outcomes Measurement Information System 29-Item Profile, reinforcing previous evidence of its effectiveness in treating lumbar spinal stenosis [172].

Another MIS approach, percutaneous pedicle screw fixation, enables spinal stabilization without the need for large incisions [173]. This technique involves inserting pedicle screws and rods through small incisions using fluoroscopic or navigation-guided systems, reducing muscle damage compared to traditional open fixation [174,175]. Hussein *et al*. evaluated 20 patients who underwent percutaneous pedicle screw fixation and found that it provided effective short- and mid-term biomechanical stabilization comparable to the conventional posterior muscle-stripping technique. Although this method has a steeper learning curve, its improved accuracy and faster recovery make it a valuable alternative to open fixation techniques [176].

### Direct pars repair

Direct pars repair is a surgical technique primarily used to treat spondylolysis and select cases of isthmic spondylolisthesis, aiming to stabilize the affected segment while preserving spinal motion. This approach is particularly beneficial for younger, active patients, as it avoids the need for spinal fusion, reducing the risk of adjacent segment disease (ASD). Over time, various techniques have been developed, evolving from traditional bone grafting to more advanced fixation methods [177-179].

The earliest direct repair technique was introduced by Kimura, who utilized bone grafting to bridge the pars defect [180]. Scott later refined this method by incorporating wiring for proper stability, followed by Buck’s technique, which involved placing a lag screw across the defect to promote fusion [181,182]. More recent advancements have led to pedicle screw-based fixation methods, offering improved biomechanical stability and better fusion outcomes [183]. Minimally invasive techniques have also emerged, reducing surgical trauma and facilitating faster recovery [184].

A meta-analysis comparing different direct repair methods concluded that pedicle screw-based techniques provide the highest fusion rates while maintaining the lowest complication rates. These findings support this method as an option in treating spondylolysis and low-grade spondylolisthesis, as they offer a balance between stabilization and motion preservation [185].

### Complications and risk factors

Complication rates after surgery for spondylolisthesis vary widely in the literature and are influenced by multiple factors, including the underlying etiology, degree of initial slippage, surgical technique, type of instrumentation, adherence to rehabilitation, and patient-specific variables [186]. A large retrospective study of 10,242 adults by Sansur *et al*. found that complication rates were higher in older patients (>65 years) and increased with the severity of spondylolisthesis [187].

One of the most frequently reported complications is pseudarthrosis, with incidence rates varying significantly between studies, ranging from none to as high as 39% [186,188]. Pseudarthrosis appears to be more common in patients undergoing surgery for isthmic spondylolisthesis compared to degenerative cases [189]. Neurological complications, including nerve root injury, radiculopathy, and cauda equina syndrome, may occur due to excessive nerve retraction, misplaced hardware, or postoperative hematoma. Patients may experience persistent pain, numbness, or weakness, and in some cases, cerebrospinal fluid leakage from dural tears that may lead to headaches and neurological irritation [190,191].

ASD is another notable complication, occurring when increased stress on levels adjacent to the fused segment leads to accelerated degeneration. This can result in new-onset disc herniation, facet joint arthritis, or spinal stenosis, manifesting as back pain, radiculopathy, or neurogenic claudication [192]. A retrospective study by Booth *et al*. analyzing 36 patients with degenerative spondylolisthesis treated with instrumented fusion found that 13.89% developed symptomatic ASD, while 19.4% had radiographic evidence of ASD without symptoms after 6.5 years [193]. Comparisons between different fusion methods suggest that PLIF may have a greater negative impact on adjacent segment degeneration than TLIF, as concluded in a study by Jiawen Ye *et al*. [194].

Additionally, reoperation rates remain a concern. A meta-analysis by Chen *et al*. reported that approximately 10% of patients require reoperation following lumbar spine surgery, with risk factors including obesity, diabetes, and smoking. These findings highlight the need for careful patient selection, surgical planning, and long-term follow-up to minimize complications and optimize outcomes [195].

## POSTOPERATIVE REHABILITATION AND LONG-TERM MANAGEMENT

### Early vs. delayed mobilization

Rehabilitation following surgery for spondylolisthesis is important for optimizing recovery and functional outcomes. The timing of early versus delayed mobilization has been a subject of research, with studies exploring its impact on patient recovery.

Early mobilization, often initiated within the first-day post-surgery, is a key component of enhanced recovery protocols. Evidence suggests early mobilization can reduce postoperative complications, shorten hospital stays, and improve functional outcomes. A narrative review suggested that early mobilization might decrease the length of stay and complication rates, although the optimal protocol specifics remain undetermined [196].

Conversely, some studies have examined the effects of delayed rehabilitation. A randomized controlled trial comparing rehabilitation initiation at 6 weeks versus 12 weeks post-lumbar spinal fusion found that earlier rehabilitation was more costly and less effective in terms of functional disability outcomes [197].

These findings suggest that while early mobilization can be beneficial, the timing and intensity should be tailored to individual patient factors and surgical specifics.

### Long-term follow-up

Long-term follow-up is essential to monitor the durability of surgical outcomes and identify potential late-onset complications. A study with an average follow-up of 11.8 years post-instrumented lumbar fusion reported a non-significant deterioration in clinical outcomes over time, indicating sustained benefits of the surgery [198].

Regular follow-up appointments, typically every three months during the first postoperative year, are recommended to ensure proper healing and spinal stability. Imaging, such as X-rays, are often utilized during these visits to assess the fusion status and detect any issues early [199,200].

## FUTURE DIRECTIONS AND EMERGING TECHNOLOGIES

Advancements in spondylolisthesis management are rapidly evolving through innovations in imaging, surgical techniques, regenerative medicine, and personalized treatment approaches. AI-assisted imaging enhances diagnostic accuracy and predicts slip progression, while quantitative MRI allows for improved assessment of disc degeneration and nerve involvement [201,202]. In surgical setup, 3D-printed patient-specific implants are being developed to optimize fusion, and navigation-assisted robotic surgery improves precision and reduces complications [203,204].

Regenerative medicine is also becoming popular, with stem cell-based therapies showing potential for disc regeneration and growth factor applications enhancing spinal fusion rates [205-208]. Additionally, personalized medicine approaches, including genetic and biomechanical profiling, are being explored to personalize treatment strategies to individual patient needs, optimizing outcomes and minimizing complications. These emerging technologies are preparing the way for more effective, customized, and less invasive treatment options for patients with spondylolisthesis [209-211].

## CONCLUSION

In conclusion, in the management of spondylolisthesis, non-surgical approaches remain the first-line treatment for low-grade cases and patients without severe neurological deficits, focusing on pain control, physical therapy, and activity modification. When conservative measures fail or when instability and nerve compression progress, surgical intervention becomes necessary, with fusion often preferred over decompression alone in cases of significant instability. In the absence of signs of instability, the debate remains on whether decompression alone is better than decompression with fusion. Long-term follow-up is essential to monitor for complications and ensure proper patient evolution. Future studies should focus on optimizing treatment selection by reaching a consensus on the indications for a specific treatment in areas of ongoing debate.
